# Inferring Gene Dependency Network Specific to Phenotypic Alteration Based on Gene Expression Data and Clinical Information of Breast Cancer

**DOI:** 10.1371/journal.pone.0092023

**Published:** 2014-03-17

**Authors:** Xionghui Zhou, Juan Liu

**Affiliations:** School of computer, Wuhan University, Wuhan, China; University of Erlangen-Nuremberg, Germany

## Abstract

Although many methods have been proposed to reconstruct gene regulatory network, most of them, when applied in the sample-based data, can not reveal the gene regulatory relations underlying the phenotypic change (e.g. normal versus cancer). In this paper, we adopt phenotype as a variable when constructing the gene regulatory network, while former researches either neglected it or only used it to select the differentially expressed genes as the inputs to construct the gene regulatory network. To be specific, we integrate phenotype information with gene expression data to identify the gene dependency pairs by using the method of conditional mutual information. A gene dependency pair (A,B) means that the influence of gene A on the phenotype depends on gene B. All identified gene dependency pairs constitute a directed network underlying the phenotype, namely gene dependency network. By this way, we have constructed gene dependency network of breast cancer from gene expression data along with two different phenotype states (metastasis and non-metastasis). Moreover, we have found the network scale free, indicating that its hub genes with high out-degrees may play critical roles in the network. After functional investigation, these hub genes are found to be biologically significant and specially related to breast cancer, which suggests that our gene dependency network is meaningful. The validity has also been justified by literature investigation. From the network, we have selected 43 discriminative hubs as signature to build the classification model for distinguishing the distant metastasis risks of breast cancer patients, and the result outperforms those classification models with published signatures. In conclusion, we have proposed a promising way to construct the gene regulatory network by using sample-based data, which has been shown to be effective and accurate in uncovering the hidden mechanism of the biological process and identifying the gene signature for phenotypic change.

## Introduction

In order to understand the regulatory mechanism in biological process, it is important to identify gene regulatory network (GRN) by using gene expression data. A lot of methods have been proposed to solve this problem, such as Bayes network [1], [2], Boolean network [3], differential equations [4], linear programming [5] and regression [6]. However, the performances of all these methods are still unsatisfactory [7].

These days, many approaches based on mutual information (MI) from information theory [8]–[12] are successfully applied in network construction [7], [13]. The information-theoretic approaches are widely used to infer GRNs because of two advantages. One is that MI can measure nonlinear dependency relations and the other one is that it can deal with a lot of variables with a few samples [7]. However, most of the existing methods establish the gene-gene causal relations by only considering gene expression levels without including phenotype information in their calculations. In other words, almost former methods just pay attention to identify whether one gene directly controls the expression of another gene [14], [15] instead of identifying their regulatory relation underlying the phenotypic alteration. Although some methods do use phenotype information to select genes related with phenotypic changes and then establish the regulatory networks, they may still ignore those genes that play critical roles in the biological process yet are not significantly correlated with the phenotype. As a result, the existing methods produce GRNs that can only reveal the static regulatory relations, rather than identify the gene dependency relations according to the phenotypic change, while the later is essential for us to understand the biological mechanism hidden behind the expression data. Although constructing the dynamic GRNs based on time-course data may reveal the regulatory relations in the dynamic process [16], the lack of time-course data and the small size of samples available make such methods inapplicable in practice.

Gene dependency is common in biological process. For example, the activities of many transcription factors regulating their targets depend on other modulators [17]; MYC activates ATM to promote apoptosis and suppress tumorigenensis [18], [19]; whether RAF1 influences the cancer prognosis or not relies on HRAS [20]. Thus if we can identify all gene dependency pairs underlying specific phenotypic change, we can better understand the biological mechanism related to the state change of the phonotype.

In this work, we propose a novel method to construct the gene dependency network underlying the alteration of the phenotype, by integrating the phenotype information and the gene expression data. We first apply conditional mutual information (CMI) to identify the gene dependency pairs underlying the phenotypic change (Each pair indicates that the mutual information of one gene and the phenotype is dependent on another gene). We then use permutation test to select the significant pairs. Finally we construct the gene dependency network by combining all pairs with significant gene dependency relationships.

Breast cancer has been widely researched and many multi-gene markers [21]–[23], also called as gene signatures, have been detected for the prediction of distant metastasis risks of cancer patients. However, the performances of most published signatures on independent data sets are very poor, even not significantly better than random signatures with the same sizes [24]. What is more, gene signatures detected by different methods may be little common, which makes the identified signatures not robust across different data sets and platforms. For example, Chuang et al. have found that the well known 70-gene signature and 76-gene signature for breast cancer have only three common genes [25]. All above phenomena may be due to the fact that tumor cells often have far more ‘passenger signals’ than ‘drivers’, and most genes of these signatures tend to be ‘passengers’ instead of ‘drivers’ [26], resulting in the lack of stability of the signatures across different data sets. For more effective therapy purpose, it is essential to identify the discriminative prognostic genes that can signal the cancer metastasis and are stable across various data sets. Now that the gene dependency network reflects the gene regulatory relations during specific phenotypic change, it is expected to be a suitable candidate to characterize the complex mechanism in the metastasis process of breast cancer. Intuitively, we think the hub nodes in the network are much more likely to be drivers instead of passengers. Thus the discriminative hub nodes from the network may be good signature genes for distinguishing the phenotype states of breast cancer.

In this work, we first construct the gene dependency network of breast cancer by integrating the gene expression data and the corresponding metastasis clinic information. Then we functionally analyze the hub nodes in the network to investigate their relations with breast cancer and cancer metastasis. Finally, we select the discriminative hub genes to form the signature to distinguish the distant metastasis risks of breast cancer patients in six breast cancer data sets.

## Materials and Methods

### Data sets and pre-processing

Gene expression data sets of breast cancer and the corresponding clinic information were downloaded from NCBI (National Center for Biotechnology Information Gene Expression Omnibus) with accession number GSE2034 [21], GSE1456 [27], GSE3494 [28], GSE4922 [29], GSE7390 [30], GSE11121 [31] and GSE12093 [32]. All these data sets have been normalized with the algorithm MAS5 and the probes have been mapped to Entrez Gene ID and averaged. GSE2034 was used for the construction of the gene dependency network as well as the selection of the signature genes, and the other data sets were used as the independent test sets. All information of these data sets is shown in Table S1.

In order to construct the gene dependency network, we first discretized the clinic information and the gene expression levels. If the distant metastasis occurred within 5 years, we set the phenotype status as 1; if the distant metastasis occurred after 5 years, we set the phenotype status as 0; the other data were abandoned. The gene expression value was set to 0 if it was lower than the median of the gene expression levels of all samples; otherwise, it was set as 1.

The human protein interaction data was downloaded from HIPPIE (Human Integrated Protein-Protein Interaction rEference) [33]. And the breast cancer related genes were obtained from the database DGA (Disease and Gene Annotations) [34].

### Inference of gene dependency network

**Figure 1:**
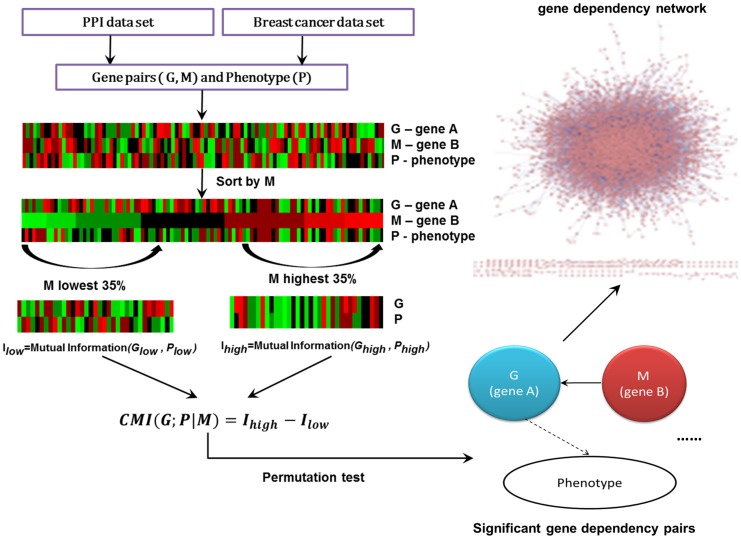
The main framework to construct the gene dependency network.

Figure 1 describes the procedure of establishing the gene dependency network. We first used CMI to infer the gene pairs with dependency relationships. Then we used the permutation method to test the significance of every gene pair. At last, we constructed the gene dependency network by combining all significant gene pairs.

#### Identification of gene dependency pairs

Our work aims at identifying the gene dependency pairs such that the relationship between one gene (denoted as A) and the phenotype (the clinic information of breast cancer patients in this work, also called as outcome and denoted as P) depends on another gene (denoted as B). In this case, we say that gene A depends on gene B in the context of phenotype P. In this work, we used CMI, calculated by the way similar to [17], to infer such dependency relationship between gene A and gene B. In order to make the calculation results more reliable, we only considered those gene pairs interacting with each other in the PPI network as candidates. For every candidate pair (A, B), we got 286 triplets in the form of (value of gene A, clinic information, value of gene B), each triplet for one sample in GSE2034. We sorted all the triplets in ascending order by the expression levels of gene B and then discretized the gene expression levels of gene A and the clinic information (Section ‘Data Sets and pre-processing’). Similar to [17], we calculated MI between gene A and the phenotype according to the bottom 35% triplets with the lowest expression levels of gene B, denoted as 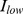. At the same time, the top 35% triplets with the highest gene B expression levels were also used to calculate MI between gene A and the phenotype, denoted as
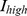
(the mutual information was calculated by a tool [35]). Therefore, the dependency relationship of gene A on gene B was calculated via the CMI described as the following equation:
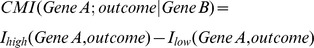



#### Significance test of the dependency relation

We used permutation method to evaluate the significance of every gene-gene dependency relation. For each candidate gene pair (A, B), we first calculated its real CMI value as described above. Then we randomly permuted the expression level of gene A and the phenotype state value across the samples 1000 times and calculated out 1000 CMI values as the null hypothesis distribution. Then the order (descending) of the real CMI value in the null hypothesis distribution divided by 1000 was taken as the significance p-value of (A,B). Finally we used a threshold (0.05 in this work) to decide whether the pair was significant or not.

#### Construction of gene dependency network

The significant gene dependency pairs were preserved to construct the gene dependency network, in which, nodes are genes, and the directed edge (B→A) represents the dependency relationship of gene A on gene B, with CMI value being the corresponding edge weight.

### Analysis of the gene dependency network

**Figure 2:**
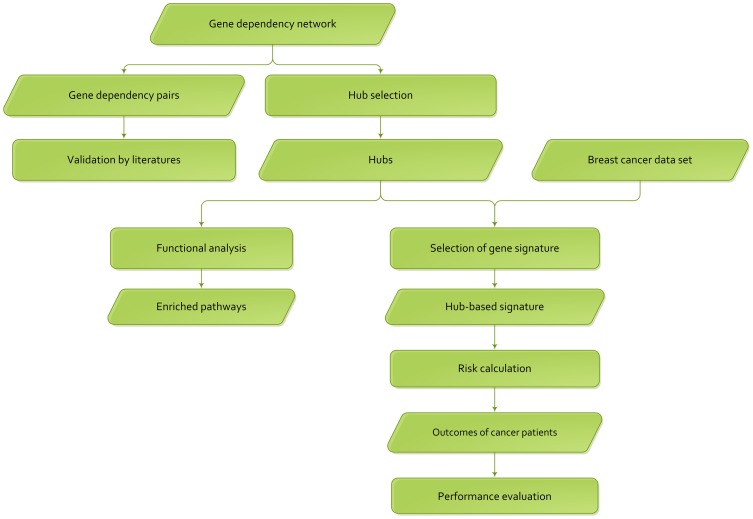
The flow chart of network analysis.

The flow chart of network analysis is shown in figure 2. Firstly, we used evidence in literature to validate the gene dependency pairs of our network. Then, we functionally analyzed the hub genes with higher out-degrees to check whether they were biologically meaningful. Finally, we selected the discriminative hubs as gene signature to predict the distant metastasis risks of cancer patients, and evaluated the performance of the selected gene signature.

#### Hub genes selection

In the gene dependency network, the out-degree of a gene indicates the number of genes related to phenotypic alteration that it influences. Therefore, the genes with higher out-degrees should be more critical to the phenotypic change. In this work, we selected the top 20% genes with the largest out-degrees (not less than 4) as hub genes of the network for further analysis.

#### Functional analysis of hub genes

In order to find out the biological functions of hub genes, we used DAVID [36] to check the significance of the overlap between hub genes and genes in each KEGG pathway. If the overlap is significant (Benjamin FDR less than 0.05), the corresponding KEGG pathway is considered to be the functional annotation of the hub genes.

#### Selection of gene candidates for signature

To select the genes significantly related to the distant metastasis risks of the patients, we adopted the strategy similar with ScoR [32] in this work. Firstly, 75% of all 286 samples in GSE2034 were randomly chosen. After that, Cox proportional hazards regression was applied to estimate the coefficient between each gene and the distant metastasis risk across the chosen patients. The procedure was repeated 400 times and the genes with Cox p-value<0.05 in more than 90% of the 400 runs were regarded as the gene candidates. As a result, 442 genes have been identified out in this way, and the final Cox coefficient and Cox p-value of each selected gene were set as the average values of 400 runs.

#### Calculation of the distant metastasis risk

In order to evaluate the distant metastasis risk of a patient, we calculated the Risk Score for each patient based on following formula by using the determined signature genes. This method is similar to the strategy of Gene expression Grade Index (GGI) [37]:
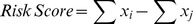
where *x_i_* (or *x_j_*) represents the expression level of the gene i (or gene j) which has positive (negative) Cox coefficient with the metastasis risk.

#### Evaluation of discrimination performance on one data set

Following the general way, we divided the patients in a data set into two groups with the same size. That is, we assigned 50% of the patients with lower risk scores into the low risk group and vice versa. After that, we used log rank test to test if the risks of the two groups were significantly different (p-value≤0.05). Kaplan Meier curves and the log rank test were performed by a tool (http://www.mathworks.com/matlabcentral/fileexchange/22317-logrank).

#### Evaluation of discrimination performance across several data sets

In order to evaluate to what extent the signature can discriminate patients with different risks in various data sets, we defined the Discrimination score (Dscore) as follows:
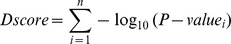



Where n is the number of the data sets, and 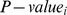 is the p-value of log rank test on the 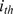 data set (Because of the problem of numerical precision, if the p-value is less than 1.00E-17, we set it as 1.00E-17). It is obvious that the larger the Dscore value is, the better the discrimination performance would be.

### Network topology and visualization

The gene dependency network was visualized by Cytoscape 2.8.2 and the topology analysis was performed by the Network Analyzer plug-in for Cytoscape [38].

## Results

### Distant metastasis-specific gene dependency network

**Figure 3:**
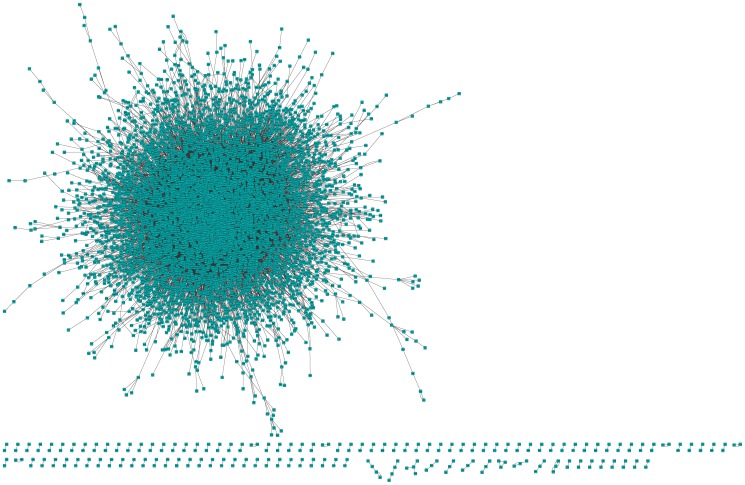
The gene dependency network. It contains 6608 nodes and 17511 edges. The edge from node A to node B means that the correlation between B and the phenotype is significantly dependent on gene A.

We used GSE2034 to infer the gene dependency pairs underlying the breast cancer distant metastasis. As a result, we got 17,511 significant gene dependency pairs (Table S2) involving 6,608 genes, and all the pairs constituted a directed network consisting of one main sub-network with 6,364 nodes and a small number of genes isolated from the main sub-network (Figure 3). The average number of neighbours (the sum of in-degree and out-degree) of the network nodes is 4.834. Moreover, both in-degrees and out-degrees of the nodes follow the power law distributions, respectively with the correlation of 0.993 and 0.976, and the R-square of 0.879 and 0.914 (Figure S1 and Figure S2). These results suggest that this network is scale free, in accordance with the characteristics of typical biological networks. It has been reported that in a scale free biological network, a few ‘hubs’ with higher degrees are more likely to be essential than others [39]. Thus, in the dependency network, a gene with larger out-degree, meaning that it can influence more genes related to the outcome of cancer patients, is more likely to have greater effect on cancer. Therefore, it is expected that the out-degree can be used to measure how much a gene can influence the distant metastasis of cancer. Moreover, experimental studies have demonstrated that only 20% nodes or so in the network are essential in influencing the state of the network [40], [41]. As a result, we selected 1,281 genes with the highest out-degrees (not less than 4), which took up about 20% of all the 6,364 genes in the main network, as the hub genes for further consideration (Table S3).

### Investigation on the hub genes

**Figure 4:**
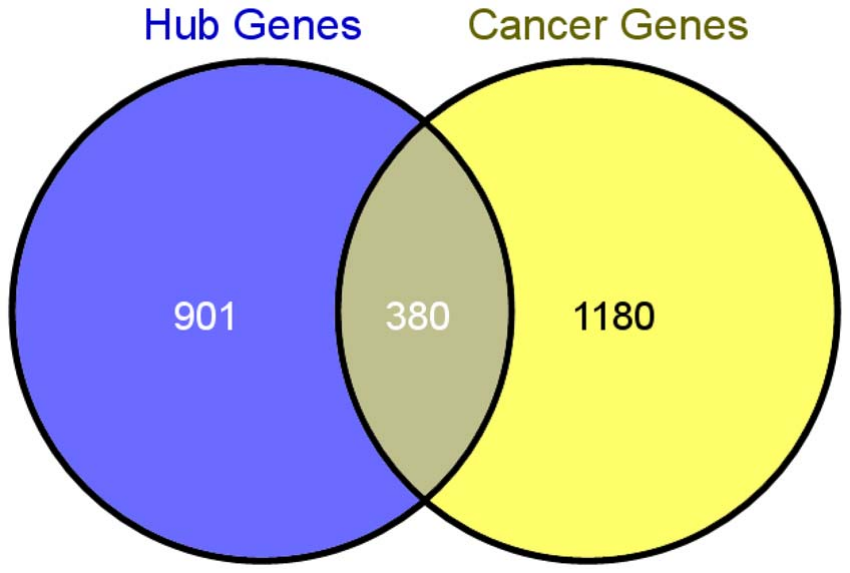
The intersection between the hub genes and the breast-cancer-related genes. Among the 1,281 hub genes in the network, 380 were reported breast cancer genes, and p-value of the intersection is smaller than 1.00E-17.

Among the 1,281 hub genes, 380 (about 30% of hub genes) genes are in the breast cancer related gene set (Figure 4), including some famous breast cancer genes such as BRCA1 (Entrez gene ID: 672), BRCA2 (Entrez gene ID: 675) [42], MYC (Entrez gene ID: 4609) [43], TP53 (Entrez gene ID: 7157) [44] and BLC2 (Entrez gene ID: 596) [45]. Using hypergeometric cumulative distribution function test, we evaluated the significance of the intersection set between the hub genes and the reported breast cancer genes, and found the overlap significant (with p-value less than 10e-17).

By enrichment analysis, 64 KEGG pathways are shown to be significant with the hub genes (with FDR less than 0.05) (Table S4). Among these pathways, ‘Pathway in cancer’ is on the top (with FDR of 1.03e-44). In addition, Cell cycle, ErbB signalling pathway, Apoptosis, Adherens junction, MAPK signalling pathway, Focal adhesion, TGF-beta signalling pathway, p53 signalling pathway, Wnt signalling pathway, VEGF signalling pathway, mTOR signalling pathway and Jak-STAT signalling pathway are all significant. In fact, all of them are the sub-pathways of the ‘Pathway in cancer’ [46], and they almost compose a whole view of ‘pathways in cancer’. It is interesting that three immune-related pathways, T cell receptor signalling pathway, B cell receptor signalling pathway and Natural killer cell mediated cytotoxicit, are also enriched with the hub genes, while immune system has been reported to have a key prognostic impact on cancer [31], [47]. Among these significant pathways, some are related with the bone, lung and brain, such as Chronic myeloid leukemia, Acute myeloid leukemia, Small cell lung cancer, Non-small cell lung cancer and Glioma, while it has been reported that these three organs suffer from metastasis most frequently in breast cancer [48]-[50]. We also found some hormones-related pathways significant, including Ubiquitin mediated proteolysis, Insulin signalling pathway, Progesterone-mediated oocyte maturation, Aldosterone-regulated sodium reabsorption and GnRH signalling pathway. In fact, hormones are associated with the metastasis risk of breast cancer and can be used as therapies for breast cancer [51], for example, progestin and GnRH are treatments for breast cancer patients [52], [53] and progesterone receptors are used as a prognostic factor in Stage II breast cancer [54]. To sum up, the hub genes are mainly enriched in four categories of pathways: ‘pathway in cancer’ and its sub-pathways, the immune-related pathways, cancer pathways related to metastasis organs of breast cancer patients (bone, lung and brain), and the hormones-related pathways.

### Investigation on the gene dependency relations

In order to validate the inferred gene dependency relations, we checked whether the relations between famous cancer genes have been reported before.

As we know, TP53 is one of the most famous cancer genes and its mutation has critical influence on cancer prognosis [55]. In the constructed network, TP53 and MYC has a significant dependency relation (with p-value of 0.048), which is in accordance with the previous finding that TP53 may induce breast cancer by stimulating MYC [54].

MYC is deregulated or overexpressed in most cancer cells and MYC inhibition is a therapy for cancer [56]. BCL2 is associated with the long term survival of breast cancer [57]. It has been reported that MYC and BCL2 act synergistically to promote primary cells into tumour cells [58], while in our work, MYC is dependent on BCL2 to influence the outcome of breast cancer (with p-value of 0.0119) and BCL2 also relies on MYC significantly (with p-value of 0.0388).

In the dependency network, another famous breast cancer gene BRCA1 has a significant dependency relation with gene JAK1 (Entrez ID: 3716) (with p-value of 0.0188), while BRCA1 has been reported to up-regulate JAK1 to govern cellular proliferation, differentiation, apoptosis and transformation; and all these processes are involved in breast tumorigenesis [59].

In conclusion, the literature has confirmed the validity of some gene dependency relations. The validation of the gene dependency relations together with the significant biological meanings of the hub genes may indicate that the gene dependency network can indeed reveal hidden mechanism of cancer metastasis.

### Distinguishing distant metastasis risks of breast cancer based on gene dependency network

To understand the metastasis mechanism, it is essential to identify gene signature that can distinguish the metastasis risks of cancer patients. Many gene signatures have been identified to solve this problem, however, few of them are satisfactory because of the poor generalization [26]. What is more, a recent report even shows that most published signatures are not significantly better than the random signatures with the same sizes [24]. The phenomenon may be caused by the fact that there are more passenger signals in tumour cells than in other types of cells and thus the real cancer genes may be burned in the gene expression profiles [26].

In the gene dependency network, a gene pair indicates that the relation between one gene and the distant metastasis risk depends on another gene. Thus, the distant metastasis-specific gene dependency network may be more likely to identify the hidden driver genes that influence the metastasis of cancer patients by modulating other passenger genes, which is very different from other methods that select the genes directly associated with the outcome. Therefore, the hub genes in the constructed network are more likely to distinguish metastasis risks of cancer patients than those selected through other methods.

#### Hub-based signature

**Figure 5:**
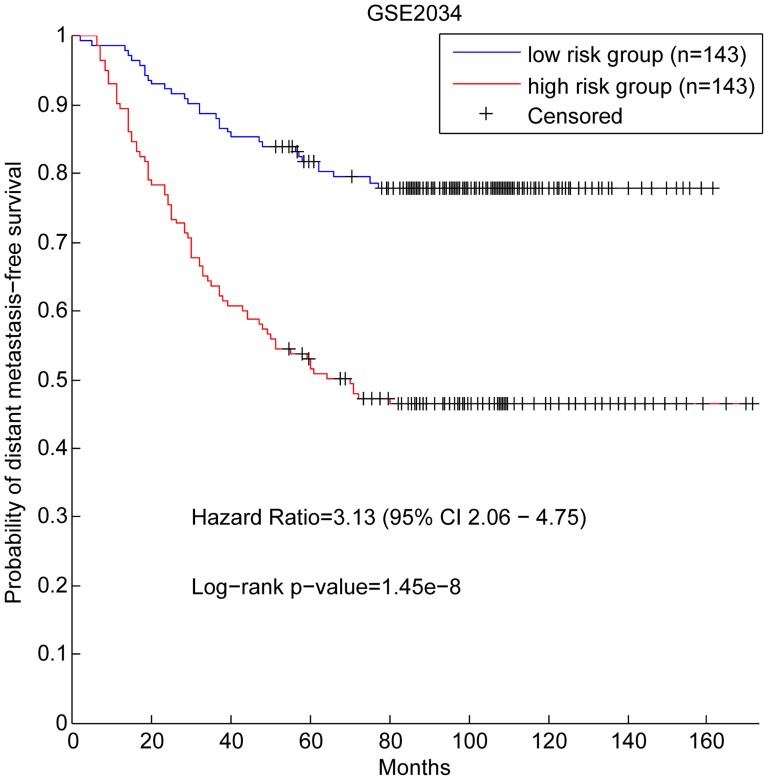
Survival analysis of the hub-based signature on GSE2034.

Among 442 genes significantly correlated with the metastasis risks (Materials and Methods) (Table S5), 43 genes (Table 1) also belong to the hub genes of the network. We therefore selected these 43 genes to form gene signature, denoted as hub-based signature, to distinguish metastasis risks of patients. The result of the survival analysis (Materials and Methods) on the patients in GSE2034 shows that the metastasis risks are significantly different between two patient groups divided based on the signature (with HR (hazard ratio) of 3.13, and p-value of 1.45e-8) (Figure 5).

**Table 1:** The hub-based signature.

Gene ID	Gene Name	Cox Beta	Cox P-value	Stability
701	BUB1B	0.46	1.07E-2	0.9625
718	C3	−0.27	1.43E-2	0.9300
890	CCNA2	0.39	8.70E-3	0.9850
891	CCNB1	0.40	1.95E-2	0.9025
960	CD44	−0.54	2.30E-3	1.0000
1917	EEF1A2	0.20	3.60E-3	1.0000
2146	EZH2	0.33	1.06E-2	0.9675
3091	HIF1A	0.44	1.94E-2	0.9150
3105	HLA-A	−0.51	1.73E-2	0.9200
3106	HLA-B	−0.39	1.06E-2	0.9600
3107	HLA-C	−0.75	2.70E-3	0.9975
3493	IGHA1	−0.39	9.72E-3	0.9675
3507	IGHM	−0.34	9.60E-3	0.9750
3659	IRF1	−0.51	1.61E-3	0.9300
3838	KPNA2	0.47	8.90E-3	0.9700
4591	TRIM37	0.58	3.90E-3	0.9950
4751	NEK2	0.38	1.80E-2	0.9200
4790	NFKB1	−0.68	6.70E-3	0.9875
4798	NFRKB	−0.78	1.53E-2	0.9300
5241	PGR	−0.13	1.64E-2	0.9300
5501	PPP1CC	1.06	8.10E-3	0.9700
5688	PSMA7	0.59	7.30E-3	0.9700
5708	PSMD2	0.67	1.97E-2	0.9100
5998	RGS3	0.66	1.02E-2	0.9650
6241	RRM2	0.37	3.80E-3	0.9975
6626	SNRPA	−0.64	1.49E-2	0.9525
6790	AURKA	0.53	2.60E-3	0.9975
6921	TCEB1	0.95	4.00E-4	1.0000
7133	TNFRSF1B	−0.41	1.37E-2	0.9525
7138	TNNT1	0.13	1.60E-2	0.9275
7289	TULP3	0.55	1.64E-2	0.9275
7936	RDBP	0.74	3.90E-3	0.9900
8445	DYRK2	0.75	2.10E-3	0.9975
8668	EIF3I	−0.70	2.01E-2	0.9025
9021	SOCS3	−0.47	1.16E-2	0.9600
9459	ARHGEF6	−0.52	3.30E-3	0.9975
10051	SMC4	0.71	2.00E-3	1.0000
10521	DDX17	−0.34	1.44E-2	0.9525
11065	UBE2C	0.43	8.70E-3	0.9650
11260	XPOT	0.65	8.90E-3	0.9725
11335	CBX3	0.85	4.30E-3	0.9925
55257	C20orf20	0.67	1.92E-2	0.9025
57122	NUP107	0.60	1.42E-2	0.9300

Elements in column 3 are the average Cox correlations in the 400 runs; elements in column 4 are the average Cox p-values in the 400 runs; the stability is the ratios of the genes which are significant in the 400 resampling runs.

**Figure 6:**
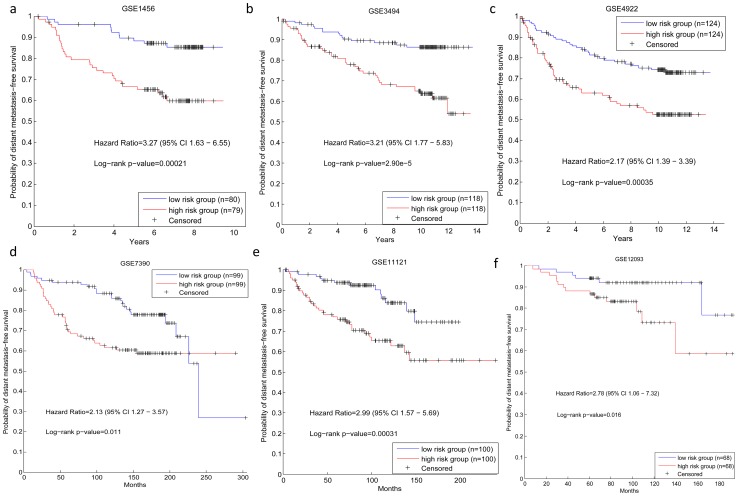
Survival analysis of the hub-based signature on six independent data sets.

To investigate the robustness of the signature, we did similar discrimination test on other six independent data sets as well. The results are shown in Figure 6. All the results demonstrate that the hub-based signature can distinguish the metastasis risks of breast cancer patients not only in the training data set, but also in the independent data sets, suggesting that the hub-based signature is superior to most published gene signatures that perform badly in the independent tests [24].

#### Comparing with pseudo signature consisting of most metastasis-risk related genes

From 442 genes significantly correlated with the metastasis risks (Table S5), we chose 43 ones that are most correlated with the metastasis risks (Table 2, denoted as pseudo signature in this work) to perform survival analysis. The results (Table 3) show that the pseudo signature cannot persistently perform well across all the data sets. More importantly, we notice that the Dscore value of pseudo signature on six independent data sets is 11.4376, which is much smaller than the Dscore value of the hub-based signature (18.9359) on the same data sets, showing that the hub-based signature performs much more stable than the same sized pseudo signature. In other words, though genes in the hub-based signature are not necessarily the most metastasis risk related genes, they are actually the ones that play critical roles in the process of phenotypic change, illustrating that the gene dependency network can uncover the biological mechanism of cancer metastasis.

**Table 2:** The pseudo signature.

Gene ID	Gene Name	Cox Beta	Cox P-value	Stability
28	ABO	−0.77	7.50E-4	1.0000
86	ACTL6A	0.69	1.50E-3	1.0000
960	CD44	−0.54	2.30E-3	1.0000
1917	EEF1A2	0.20	3.60E-3	1.0000
3537	IGLC1	−0.28	1.00E-3	1.0000
6810	STX4	−0.78	3.90E-3	1.0000
6921	TCEB1	0.95	4.00E-4	1.0000
9541	CIR	−0.89	1.40E-3	1.0000
9648	GCC2	−0.25	3.30E-3	1.0000
9675	KIAA0406	0.63	2.80E-4	1.0000
9764	KIAA0513	0.53	3.70E-3	1.0000
9837	GINS1	0.41	1.10E-3	1.0000
10051	SMC4	0.71	2.00E-3	1.0000
10057	ABCC5	0.57	4.20E-4	1.0000
10628	TXNIP	−0.50	1.10E-3	1.0000
10961	ERP29	−0.77	2.50E-3	1.0000
23167	EFR3A	0.69	1.60E-3	1.0000
23174	ZCCHC14	0.87	1.40E-3	1.0000
25793	FBXO7	−1.08	2.60E-3	1.0000
27250	PDCD4	−0.46	2.00E-3	1.0000
27350	APOBEC3C	−0.54	4.70E-3	1.0000
28813	IGLV2-23	−0.23	1.00E-3	1.0000
28814	IGLV2-18	−0.33	1.10E-3	1.0000
28816	IGLV2-11	−0.24	8.90E-4	1.0000
29035	C16orf72	−0.39	1.40E-3	1.0000
29127	RACGAP1	0.65	1.30E-4	1.0000
50802	IGK	−0.24	3.30E-3	1.0000
51110	LACTB2	0.57	8.60E-4	1.0000
51222	ZNF219	−0.68	9.80E-4	1.0000
54732	TMED9	0.59	1.50E-3	1.0000
55110	FLJ10292	0.35	1.80E-3	1.0000
55508	SLC35E3	0.41	1.60E-3	1.0000
55596	ZCCHC8	1.00	7.60E-4	1.0000
57092	PCNP	0.91	2.00E-3	1.0000
58189	WFDC1	0.44	3.10E-4	1.0000
79822	ARHGAP28	−0.45	7.30E-4	1.0000
80700	UBXD1	−0.68	1.90E-3	1.0000
118433	RPL23AP7	0.47	4.90E-3	1.0000
259266	ASPM	0.34	3.80E-3	1.0000
442334	ARF1P1	0.35	1.80E-3	1.0000
650405	LOC650405	−0.34	5.10E-4	1.0000
652493	LOC652493	−0.26	1.50E-3	1.0000
652694	LOC652694	−0.32	2.70E-3	1.0000

The 43 genes are used as the pseudo signature for comparing purposes: elements in column 3 are the average Cox correlations in the 400 runs; elements in column 4 are the average Cox p-values in the 400 runs; the stability is the ratios of the genes which are significant in the 400 runs.

**Table 3:** Performance of the pseudo signature.

data sets	HR	HR 95% CI-	HR 95% CI+	log rank p-value
GSE2034	4.65	2.95	7.33	3.14E-13
GSE1456	1.90	1.00	3.60	3.20E-02
GSE3493	2.05	1.18	3.59	5.56E-03
GSE4922	1.72	1.11	2.66	9.60E-03
GSE7390	1.76	1.06	2.93	5.40E-02
GSE11121	2.02	1.11	3.67	1.30E-02
GSE12093	3.84	1.37	10.73	2.90E-03

GSE2034 is the train data set and the other six are the independent data sets.

#### Comparing with random gene signatures

Considering most published signatures on independent test sets have been argued to be very poor, even not significantly better than random signatures with the same sizes [24], we also compared the performance of our signature with the random gene signatures of the same sizes by two ways to check whether the former was significantly better than the latters.

**Figure 7:**
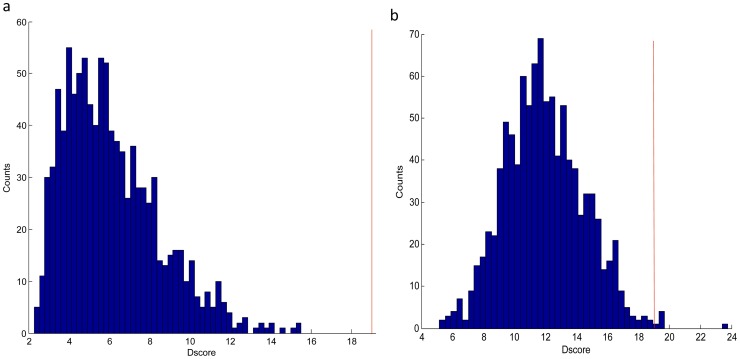
The significance test of the hub-based signature with the random signatures. a. The random signatures were randomly selected from all of the 13,698 genes; b. The random signatures were randomly selected from the 442 genes that are significantly correlated with the metastasis risk. In both sub-figures, the red line is the Dscore of the hub-based signature (18.9359), and the blue bars are the distribution of the 1,000 random signatures' Dscores.

In the first test, we randomly selected 43 genes from the whole gene set (13698 genes in total) to form a random signature, and the patients from each of the six independent data sets were divided into good and bad prognosis groups according to the metastasis risks computed via the random signature, based on which Dscore was calculated. The above procedure was repeated 1000 times to obtain 1000 Dscore values. As a result, we found the Dscore followed a chi-squared distribution (Figure 7a), which is conform to the definition of the Dscore. Therefore, we used the Fisher's combined probability test to evaluate the significance of the hub-based signature' distinguishing capability and got the p-value approaching to zero (p-value less than 1.00E-17).

In the second test, we randomly selected 43 genes from 442 genes that were significantly associated with the metastasis risk (Table S5) and performed the similar procedure as above. Dscore values of 1000 runs are found to follow a Gaussian distribution (Figure 7b). Among the 1000 Dscore values, only six are not less than 18.9359, illustrating that the p-value of the significance test is 0.006. That is to way, the hub-based signature also significantly outperforms this kind of random signatures (Figure 7b).

It has been argued that most published signatures are not significantly better than the random signatures [24]. However, above two significance tests illustrate that our hub-based signature significantly outperform the random ones. The results demonstrate that the gene dependency network can reveal the biological mechanism in cancer metastasis, thus the hub genes in the network are more likely to be the driver nodes underlying the phenotypic change.

## Discussion

Although many methods have been proposed to construct gene regulatory networks, few of them investigates the gene dependency relationships specific to phenotypic change that are common in biological process. In this work, we have proposed a novel methodology to infer the gene dependency pairs underlying the specific phenotypic change. Concretely, we applied CMI to identify the gene dependency pairs, each of which indicates that the correlation between one gene and the phenotype is dependent on another gene. All the significant gene pairs are combined together to construct the gene dependency network that could characterize the gene regulatory mechanism underlying the alteration of the phenotype. When applied to sample based gene expression data of breast cancer (with distant metastasis or non-distant metastasis clinic information), the network has been demonstrated to be able to uncover the biological mechanism in the metastasis process. The hub genes in the network have significant intersection with the breast cancer related genes, and the functional analysis shows that the hub genes in the network are biologically significant. Moreover, many gene dependency relations reported previously in literature can be detected in the network. Furthermore, the hub-based signature can distinguish the cancer prognosis of breast cancer patients with robust performances across various independent data sets, which is significantly superior to most published gene signatures that have been argued to perform badly in the independent tests and even not to be significantly better than the random signatures [24].

The good performance of hub-based signature may owe to the following merits of our method. First, we directly included the phenotype information in the inference of the gene dependency relations, thus the gene dependency network can reveal the gene regulatory relation during the alteration of the phenotype. Second, gene dependency network focuses on modulators that can influence the phenotype via other genes, so the network can uncover the hidden biological mechanism that may be ignored by former researches. Therefore, the hub genes modulating the largest number of genes may be the key genes influencing the distant metastasis of breast cancer and have robust discrimination capability across different data sets.

As illustrated from the results, our methodology can reveal the hidden mechanism in the metastasis of breast cancer. In fact, it can also be applied in other fields, such as the mechanism in the disease progress, embryo development, and other aspects as long as there are enough gene expression data and the corresponding phenotype information. In this work, our methodology succeeded in the binary phenotype data sets (metastasis and non-metastasis). However, it is also suitable for the data sets with more than two phenotype states.

Of course, the methodology proposed in this paper only works on the sample based data with more than one phenotype states. In the case of single phenotype state, for example, only non-metastasis samples, our method fails to work. In addition, the calculation of CMI would be inaccurate if there are not enough samples in the data set. These limitations should be addressed in future study.

## Supporting Information

Figure S1
**The power law fit of the in-degree.** 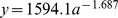. The correlation is 0.993 and the R-square is 0.879.(TIF)

Figure S2
**The power law fit of the out-degree.** 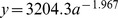. The correlation is 0.976 and the R-square is 0.914.(TIF)

Table S1 **Data sets used in this work.** All the data sets are available at NCBI GEO.
(XLSX)

Table S2 **All significant gene dependency pairs.** All the gene pairs in the table are with p-values less than 0.05. Genes in the first column are the modulators of the gene pairs.
(XLSX)

Table S3 **The selected hubs in the network.** The genes with the highest out-degrees are selected as hubs (20% of all the genes).
(XLSX)

Table S4 **Enriched pathways.** The significant enriched pathways in the table are with Benjamini FDRs less than 0.05.
(XLSX)

Table S5 **All the candidate genes which are significant with the metastasis risk of breast cancer.** A gene is selected as a candidate when it is significant in 90% of the 400 runs.
(XLSX)
