# Supplementary figures and images for: Inferring Gene Dependency Network Specific to Phenotypic Alteration Based on Gene Expression Data and Clinical Information of Breast Cancer
Source: PLoS One. 2014 Mar 17;9(3):e92023. doi: 10.1371/journal.pone.0092023 (PMC3956890; doi:10.1371/journal.pone.0092023)

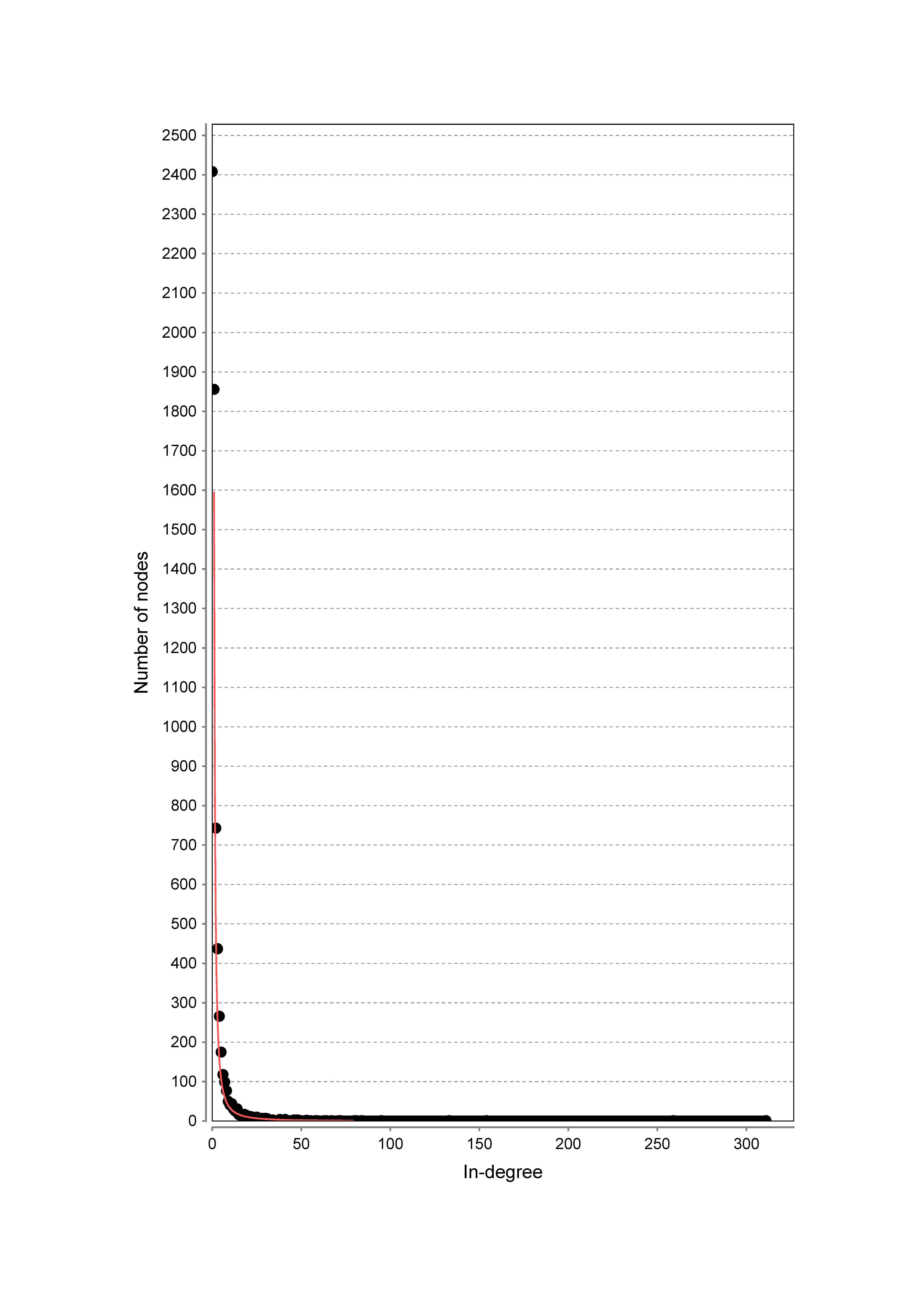

Supplement: Figure S1 — The power law fit of the in-degree. . The correlation is 0.993 and the R-square is 0.879. (TIF) [file pone.0092023.s001.tif]

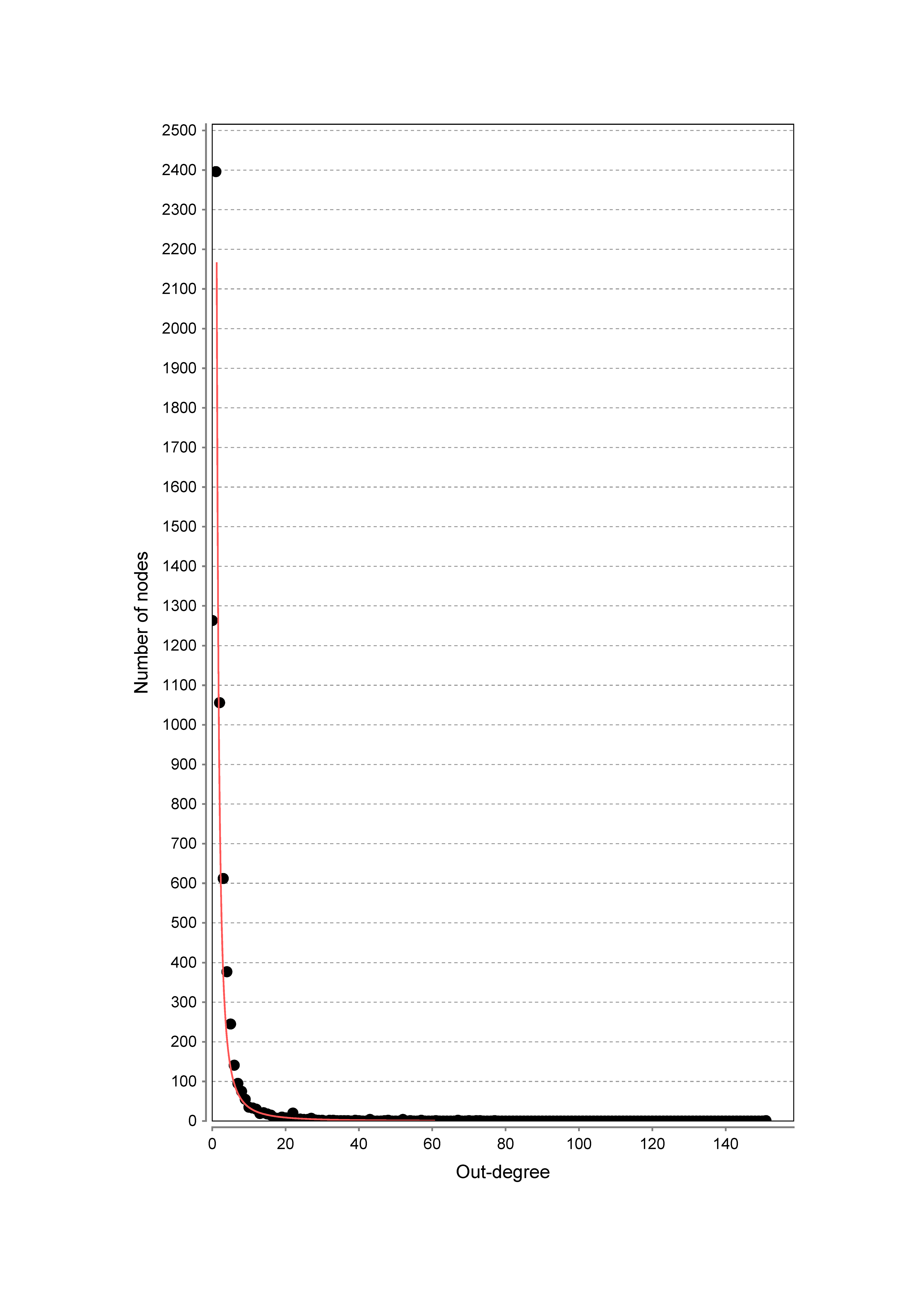

Supplement: Figure S2 — The power law fit of the out-degree. . The correlation is 0.976 and the R-square is 0.914. (TIF) [file pone.0092023.s002.tif]
